# A conceptual analysis of maltreatment in sports: A sport social work perspective

**DOI:** 10.3389/fspor.2022.1017308

**Published:** 2022-11-03

**Authors:** Courtney Gattis, Matt Moore

**Affiliations:** ^1^College of Social Work, Florida State University, Tallahassee, FL, United States; ^2^Department of Social Work, Ball State University, Muncie, IN, United States

**Keywords:** athlete, trauma-informed, maltreatment, abuse, therapy, sport social work

## Abstract

Maltreatment in sports is an epidemic and occurs in many relational forms such as physical, sexual, and emotional abuse or neglect. Maltreatment in sports also exists in forms of non-relational abuse and focuses on mezzo and macro forms of maltreatment such as systematic abuse, organizational abuse, and physiological abuse or neglect (e.g., exploitation and athlete trafficking). It is imperative to study the effects of maltreatment in sports as athletes can be the victims (survivors) as well as perpetrators of abuse. Recent research in the field of social work points to the benefits of Trauma-Informed Sports and Short Focused Brief Therapy (SFBT) as possible interventions. It is imperative for transferability in the field of maltreatment in sports, that practitioners define each form of abuse in the context of maltreatment and trauma. This commentary aims to discuss the different forms of maltreatment in sports that could affect athletes and provide discussion and insights into the void of research surrounding certain forms of non-relational abuse and the role trauma-informed therapies serve in promoting athlete wellbeing from a social work lens.

## Maltreatment in sports: A sports social work perspective of athlete abuse

Maltreatment in sports is an undeniable problem. Athletes might experience sexual maltreatment, physical maltreatment, psychological maltreatment, neglect, and exploitation in practicing their sport (1, 2). Mountjoy (3) found 46% of athletes are unaware of the types and extent of abuse they face in sport. Athletes are particularly vulnerable to maltreatment due to the unique characteristics of the sports environment. These characteristics include the importance placed on the coach-athlete relationship, the intensity of the sport, the demands of competition, the interest of the media, time spent in distant training centers, recruitment procedures of young athletes, and the distance from an athlete's home or school environment (4).

This conceptual analysis aims to discuss the current void of research in sports maltreatment from a social work and trauma-informed perspective. The analysis defines and discusses examples of relational and non-relational forms of maltreatment in sports. The piece also discusses athlete health and wellbeing, a culture of safety in sport, and trauma-informed interventions from a social work perspective. The authors provide future recommendations for advancing the assessment and intervention process of athletes experiencing maltreatment. This conceptual analysis provides a timely review of risk and safety concerns in the world of youth, collegiate, and professional athletics. Many of these risk factors could impact athletes to a greater degree given the post-pandemic challenges faced in social and emotional development (5). This is a time where athletes need coaches and other mentors in their lives helping them to reintegrate back to the pre-pandemic world and to make up for lost gains as opposed to putting athletes at further risk of maltreatment (6).

## Types of maltreatment

Athlete vulnerability concerns arise when the sport puts individuals at risk for abuse and exploitation. Researchers categorize maltreatment as relational and non-relational in nature. Since most forms of maltreatment exist within the context of relationships, it is imperative to understand the nature of relationships that exists within an athletic setting. Additionally, athletes may experience multiple forms of abuse and maltreatment due to these complex relationships (1, 2).

### Relational maltreatment

Relational maltreatment typically includes four types of abuse: physical, sexual, emotional, and neglect (7, 8) (see Table 1). These forms of relational maltreatment occur within the context of a critical relationship that significantly influences an individual's sense of safety, trust, and fulfillment of needs (7, 8). These relationship roles within the sports contexts can include the relationship between members of the athlete's entourage such as teammates, coaches, support staff, spectators, family, etc. (14).

**Table 1:** Relational maltreatment.

Physical abuse Physical abuse is defined as being deliberately hurt by an adult person causing injuries such as bruises, broken bones, burns or cuts (9).	Sexual abuse Sexual abuse in sport includes having a sexual relationship with an athlete; improper sexual contact, including genital penetration; indecent exposure; providing rewards for sexual favors; sexually explicit comments, jokes, or gestures; sexual propositions; and showing an athlete pornographic images, videos, or other material (10).	Emotional abuse In sports contexts, emotional abuse must represent a pattern of deliberate, non-contact behaviors within a critical relationship that can potentially be harmful and can be seen as belittling, humiliating, shouting, and threatening (8).	Neglect Neglect is characterized by an omission of care such as withholding food or nutrition and forcing to train while injured (11).
• Overtraining • Pushed, shoved or shook you up • Thrown something at you • Hurt you with her/his hands • Slapping • Hitting • Shaking • Throwing equipment at or near a player • Kicking • Pulling hair or ears • Striking • Shoving • Grabbing • Hazing • Punishing “poor” play or rule violations through the use of excessive exercise • Punishing “poor” play or rule violations by denying fluids • Punishing by the denial of sustenance or food • Rough physical corrections of position/movement	• Sexist jokes and remarks (12) • Intrusive sexual glances • Sexually explicit communication • Requesting sexual acts • Indecent exposure • Fondling genitals or breasts • Penetration • Rape • Sodomy • Sexual exploitation • Exposure to pornographic materials • Creation of child sexual abuse materials	• Denial of attention or support, such as intentionally ignoring an athlete for poor performance (13) • Name calling • Insulting • Shouting • Belittling • Threatening • Humiliating • Scapegoating • Ignoring • Rejecting • Bullying • Taunting • Shunning • Isolating • Gaslighting • Quick oscillation between praise and criticism • Refusing to give corrections to the athlete • Promoting disordered eating	• Omissions of care and in sport can be exhibited by failing to provide adequate injury care or supervision • Improperly treating injuries and forcing injured athletes to play • Inadequate equipment • Lack of supervision during overnight trips • Allowing bullying or hazing by teammates • Not having basic needs met such as food/water

### Non-relational maltreatment

Non-relational abuse occurs in non-critical relationships. These types of abuse include systematic abuse, organizational abuse, and physiological abuse (see Table 2). While the abuse may still be perpetrated by a coach, teammate, or parent, it is imperative to distinguish whether the relationship is critical or non-critical to the athlete victim (survivor). Examples of non-critical relationships may include the athlete and universities, athletic directors, team owners, team doctors, and fans. Whether relational or non-relational both types of maltreatment impact an athlete's wellbeing.

**Table 2:** Non-relational maltreatment.

Systematic abuse Systematic abuse involves organizational and sociocultural influences, such as sport philosophies, a lack of strong athlete protection measures, and a lack of enforcement and accountability, by authority figures in key organizations critical to fostering an environment that allows athletes' concerns to be ignored (15)	Organizational abuse The historical power of autonomy and self-governance afforded to sport organizations contributes to fragmentation and a general lack of monitoring and accountability, thus allowing issues such as maltreatment of youth athletes to be perpetuated and even legitimized (8, 15)	Physiological abuse Physiological maltreatment is considered behaviors that promote corruption, isolation, exploitation, and the adoption of destructive, antisocial, or unhealthy behaviors (4).
• Institutional barriers including a culture of fear and silence, lack of trust in sport organizations around the handing of safe sport issues, and fear of repercussions for reporting (11) • Sport administrators minimizing and trivializing reports of abuse from victims, blaming the victims for the abuse experienced, and challenge the credibility of an athlete to defend the abuse (11) • Investigations obscured by administrators' failures to investigate, inadequate investigations that are slow and kept quiet, a lack of appropriate action when a legal duty to report is necessary, and collusion between administrators and other stakeholders to minimize impacts or silence victims (11) • Systemic violence that ‘is enabled, tolerated and, in some instances, actively encouraged' by the organizational structures of sport with factors that include “(a) structural factors (i.e., power imbalance, winner-take-all rewards, isolation), (b) social factors (i.e., conformity to dominant values, perceived instrumental effects, organizational tolerance), and (c) organizational stressors (i.e., role conflict and ambiguity, depersonalization, intensification, deficient internal communication, professional uncertainty)” (16)	• A culture and disciplinary structure wherein the abusers would be sheltered from justice, and the abused would be silenced (17) • Abuse from spectators, discrimination, cultures which normalize abuse, unhealthy training programs, hazing, medical mismanagement, systematic doping and age-cheating (18)	• Forcing athletes to excessively train beyond reasonable physical and mental exhaustion • Forcing to train while injured despite medical advice • Forcing to attempt movements outside of one's ability putting them at risk of severe injury • Parental isolation • Physical scrutiny in front of team members • Harassment, derogatory comments by a superior • Bullying and spreading rumors about teammates
**Exploitation:**		**Trafficking:**
• Athletic social reproductive labor, like all forms of social reproductive labor, arrives as labor through the intersectional forms of structural power, privilege, and marginalization that shape our global political economy (19). • Whether professional or at the elite U.S. college level, it seems clear that athletic social reproductive labor in many (but not all) sporting contexts falls disproportionately to Black athletes tasked with reproducing White spectatorial subjects (19). • Some athletes are treated like commodities with their ‘price’ being estimated by sports teams and sponsors according to their entertainment value and TV viewers' demands. • To maximize the career income of athletes, some sport management groups do not hesitate to scour the world to detect and contract potential talents (20).		• Mainly takes the form of adolescent athletes trafficked and sold in the context of transboundary, illegal transfers between clubs (20) • Most types of slavery, the sale and trafficking of athletes mainly occurs in situations of economic exploitation where those with an advantage – often economic – over others use their power to impose unfair practices. In sports, as in other domains, financial power is concentrated in the hands of North Americans and Western Europeans (20). • When children are sold to rich football or baseball teams, their passion for the sport is at the origin of the criminal activity. Unscrupulous traders often rely on the fact that young athletes and their families are desperate to join a major sports team and have a lucrative career in the industrialized world, which complicates the prevention of trafficking and sale in sports (20).

## Athlete health and wellbeing

Examining the impact of athlete maltreatment through a biopsychosocial perspective can deepen our understanding of the complexities of sport-related maltreatment and how it affects an athlete. The biopsychosocial model emphasizes a holistic health perspective in which biological, psychological, and social health symptoms and conditions are causally influenced by each other (21).

### Biological impacts of maltreatment

Biological effects of maltreatment impact individuals throughout their life span. Repeated stress and trauma impact brain development and functioning, which control regulatory responses and processes of the autonomic nervous system leading to increased degeneration of neural processing known as the allostatic load (21). The allostatic load impacts the hippocampus, an integral part of the limbic system, a cortical region that regulates motivation, memory, emotion, learning, and memory (22).

Scientific research shows adults who experience maltreatment have lessened gray matter volume in their hippocampus and increased gray matter volume in the dorsomedial prefrontal cortex and the orbitofrontal cortex, which are emotional and mental regulator systems and increase their risk of developing major depressive disorder (21). This may be an important element to further research as post-mortem chronic traumatic encephalopathy (CTE) diagnoses also reflect changes in gray matter associated with head trauma due to sports.

Detrimental effects on the autonomic nervous system due to maltreatment include decreased restricted cardiac output activity and dysregulated patterns of physiological reactivity (23). In sports contexts, a “challenge state” characterizes an increased cardiac output, which research shows a positive association with performance (24).

Dysregulated cortisol levels and dehydroepiandrosterone (DHEA-S), or androgen, correlate with multiple health issues like high blood pressure, mood changes, weight gain, and overactive adrenal gland responses (21). Androgen is a male-type hormone produced in males and females but is commonly higher in males. These levels can escalate when individuals experience chronic psychosocial stress (21). Low energy availability is also associated with certain forms of maltreatment, including overtraining and malnourishment and is exacerbated by the mental toll of body shaming and public weighing practices (18).

### Psychological impact of maltreatment

Maltreatment can also cause severe negative impacts on the psychological wellbeing of athletes. Various forms of maltreatment may impair performance, increase willingness to cheat, and lead to athletes dropping out or other psychosomatic disorders, including depression, anxiety, post-traumatic stress disorder (PTSD), disordered eating, substance abuse, self-harm, and suicide (11, 18, 25, 26). Depression, anxiety, and PTSD can have long-term effects and present years after retirement in athletes (26–29).

Female athletes are more vulnerable to adverse effects of maltreatment. Elite female athletes who experience emotional abuse often experience social withdrawal by the end of their careers (13). Traumatic correlations of sexual abuse in female athletes are associated with poorer sports performance or dropout, lower self-esteem, and increased anxiety than in males. Research also shows graded relationships between polyvictimization and the severity of psychological symptoms in athletes who experience maltreatment into adulthood (26).

Athlete victims (survivors) of maltreatment may conceal their distress to maintain their team position or preserve anonymity (11). Their claims may also be silenced or denied by people of power within the sport, creating a lack of formal accountability and leading athletes to believe such behaviors are socially and legally acceptable (18, 30). This impression can exacerbate the initial psychological trauma experienced by the athlete survivor.

### Social impact of athlete maltreatment

Social relationships significantly protect individuals from several causes of morbidity and mortality, including depression and social relationships (26). Individuals with a history of maltreatment have a higher likelihood of long-term attachment difficulties, a higher risk of experiencing intimate partner violence and victimization, greater difficulty forming attachments and developing social competence, decreased life satisfaction, and a significantly lower sense of social wellbeing (21, 31, 32). Many athletes suffer performance detriments, opportunity costs, reduced medal chances, loss of sponsorship, increased willingness to cheat and may choose to quit their sport due to the impacts of maltreatment endured (18).

Sports maltreatment occurs as a broader societal and social problem, with societal risk factors based on entrenched prejudices expressed through power differences and through range of interpersonal mechanisms. Athletic identity refers to the degree of strength and exclusivity to which a person identifies with the athlete role or the degree to which one devotes special attention to sport relative to other engagements or activities in life (33). Brewer's research summarizes athletic identity functions as a cognitive structure and a social role, stemming from individual emotional connections and feedback from others, such as teammates, coaches, parents, and spectators (34). The hierarchical structure of sports, the bodily contact, the male-dominated gender ratio, the authoritarian leadership, and existing rewards systems can impact prosocial and antisocial behaviors (18, 26). The impact of maltreatment on relational functioning in younger athletes negatively influences psychological and neural development that influences attachment and relational skills (35).

Evidence on the early impacts of maltreatment supports the importance of the interaction of the parent-child relationship quality and shows that maltreatment can impact the frontal lobe volume in adulthood (21). This is especially important in sports contexts as often athletes spend as much time with the coach, sports stakeholders, or athlete entourage as they do with their parents; thus, the relationship is as crucial to healthy social development.

## Social workers in sport

**Figure 1:**
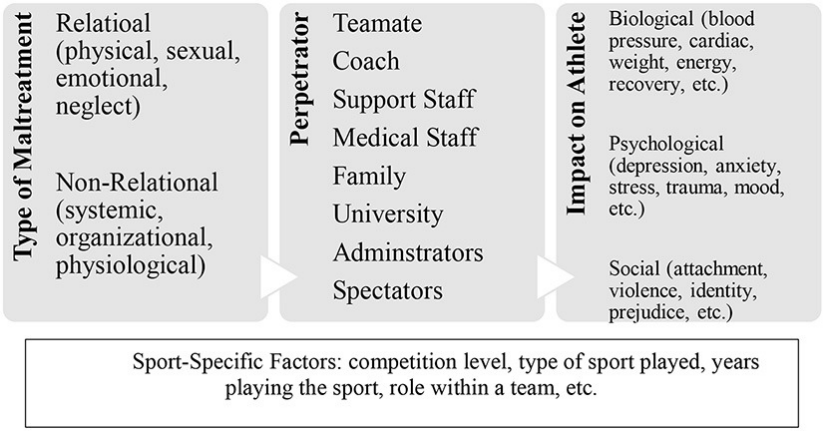
Diagram for maltreatment in sport.

Prevention of maltreatment in sports requires a multifaceted approach relative to sport-specific factors of risk and the implementation of prevention initiatives over time (8). Sport specific factors might include: competition level, type of sport played, years playing the sport, role within a team, athlete-coach relationship, the interest of the media, sport-related pressure, etc. (36). These factors combined with elements of relational and non-relational abuse create a paradigm for engaging with athletes around these traumatic experiences (see Figure 1). Social workers can serve as a vital resource as part of an interdisciplinary athletic team by implementing person-centered interventions, connecting athletes to appropriate resources, and advocating on behalf of the athlete (2). The sport social worker's role is to help athletes identify their strengths, build awareness of their body and mind (triggers, warning signs), and identify personal goals, which leads to greater autonomy. As part of the interdisciplinary team, sports social workers can provide education, foster relationships with other professionals and stakeholders, and advocate for political changes to enhance the wellbeing and safety of athletes and sports communities (37). This could also include a culture of safe sport with an emphasis on policy, coach and parent education, and work with sport administrators to define a philosophy that fosters safety and facilities positive developmental outcomes.

Sport social workers are individuals who athletes perceive as neutral and unaffected by the power structures in sport. They protect and enhance the wellbeing of athletes and assist athletes with their negotiations in relationships with others (6, 13, 36). Sport social workers are in a unique position to advocate for athletes within various sports systems and settings due to their values, knowledge, and approaches. Sport social workers are guided by a code of ethics which promotes diversity and inclusion. In sports contexts, creating diverse and inclusive practices are paramount in competitive sports at community and intercollegiate levels, where athletes or non-revenue sports may be more vulnerable to privilege and oppression (37).

Sport social workers promote the health and wellbeing of athletes through direct practice, community organizing, advocacy, policy development, education, and research on micro, mezzo, and macro levels (2). Sport social work promotes social justice and social change by focusing on the unique needs of athletes at the individual, environmental, and organizational levels (38). When an athlete or other individual reports or suspects abuse, social workers have specific training to respond to the needs of the target population.

Central concepts within social work education and practice include perspectives on biopsychosocial wellbeing, person-in-environment, and ecological systems. Sport social workers understand the importance of a holistic, rather than singular, perspective when working with athletes and that athletes develop within social environments. Holistic approaches in athlete development focuses on the athlete's physical, psychological, and social development through sports participation (2, 13, 39).

Sport social workers also recognize the critical nature of interacting with other environments and social systems. Within an ecological theoretical foundation, sport social workers consider how the athlete's past leads to various levels of vulnerability. Sport social workers seek to understand the root of the issue and how it affects the athlete's vulnerability on micro, mezzo, and macro levels (40, 41). Furthermore, sport social workers understand the context within which players live and comprehensively understand how this context and local culture(s) impact the player's understanding and experience with trauma (42).

Sport social workers also have unique training in person-centered, strength-based approaches. The ability to meet an athlete where they are allows social workers to demonstrate competence within sports and foster rapport within the settings of the sport (2). Strength-based approaches when working with athletes can foster life skills transferability promotion outside of sports contexts and promote better results with athlete performance (41). When maltreatment occurs, especially at an elite level, sport social workers can help guide athletes through these experiences. Sport social workers are trained in specific interventions, like cognitive behavioral therapy (CBT) and eye movement desensitization and reprocessing (EMDR), that can reduce psychological symptoms of the maltreated athlete (13).

Sport social workers also understand the difference between professional collaboration and professional competition (2). There is a strong desire from sport social workers to embrace the expertise of sport psychologists, performance psychologists, clinical psychologists, athletic trainers, and all other member of an athlete support team. Sport social work continues to learn not only from the competencies and values of the social work profession, but also, the long-standing history of other professions engrained in the culture of sport. This is not a matter of one profession being better suited to address athlete maltreatment than another–this is a matter of providing an integrated approach to care that supports the complexities of an athlete's life (2).

In all categories of maltreatment, research shows the higher level of sport competition increases the prevalence rate of harm compared to athletes in lower levels of competition (12). Athletes may show signs and symptoms of abuse that do not mimic traditional clinical features due to the unique nature of the competitive arena, power dichotomies within relationships in sport, and lack of athlete autonomy. Education in trauma-informed care specific to athlete culture is imperative to maximize the likelihood of identifying symptoms of maltreatment where symptoms do not mimic traditional clinical features. Sport social workers along with other care providers have training in evidence-based practices related to trauma.

## Trauma-informed sports

Sports acceptance is part of the cultural fabric. Integrating cultural competence in working with athletes is essential, as managing trauma and grief varies by culture (43–46). Adding local cultural elements can increase familiarity and safety, fostering greater sustainability (47). Sports social workers practicing through a trauma lens will provide fundamentals of therapeutic rapport, understanding the athletes' experiences, and helping the athlete understand their perception of reliance and risk (48).

Athletes may present symptoms of abuse differently than non-athletes due to sports cultures encouragement of obedience and the suppression of emotions (49). Researchers formulated the ‘protection hypothesis,' which suggests sports protect athletes from experiencing violence outside sport and may guard them against more severe consequences if they experience it (26).

In addition to CBT and EMDR previously mentioned in this commentary, other therapeutic modalities guided by trauma-informed principles may be considered, such as Dialectical Behavioral Therapy [DBT; (50)], ARC [Attachment, Self-Regulation, Competency; (51)], Solution-Focused Brief Therapy [SFBT; (52, 53)]. Parent-Child Interactive Therapy [PCIT; (54)], and Narrative Therapy (55). These approaches are skills-based focused, which is critical for athletes in identifying and emphasizing their strengths to then build efficacy and self-esteem in trauma-informed sports approaches. This is certainly not an exhaustive list of treatment modalities; however, provides a glimpse into the training received by sport social workers with a clinical expertise.

Skills- and strengths-based focuses are crucial elements in working with trauma-exposed individuals who are often overwhelmed with feelings of guilt, blame, fear, and anxiety (56–58). Dependent on the sports culture and athlete victimization, broader trauma-informed sports modalities to work with teams and organizations, rather than focusing on an individualistic approach, can also be utilized in trauma-informed sports. As sport social workers and other learn more about these evidence-based methods they can work in a transdisciplinary manner to continue efforts for creating a culture of safety within sport.

## Culture of safety

*Safe sport* is defined as an athletic environment that is respectful, equitable, and free from all forms of non-accidental violence to athletes (18). Safe sport initiatives include education, codes of conduct, athlete representation, and independent investigation practices into maltreatment investigations. The safe sport culture should also be inclusive and acknowledge that sports experiences differ depending on the groups of people (e.g., females, athletes from underrepresented minorities, para-athletes, etc.).

A safe sports culture should be focused at all ages and types of athletes with understanding risk for maltreatment is highest in elite, disabled, child, and LGBT athletes (18). Sports personnel involved in athlete development and performance have a particular responsibility to operate within professional boundaries, to understand, identify and refer signs of harassment and abuse, and to mitigate associated risks (18).

Challenges to advancing a culture of safe sport include a winning at all costs mentality, normalization of harm, lack of attention to equity, diversity, and inclusion, a culture of fear and silence, and lack of trust in sports organizations to handle safe sport issues (12). Unless specifically trained and qualified in this field, entourage members and other interdisciplinary professionals should not attempt to evaluate or treat athletes alleging maltreatment. Instead, they should refer all disclosures to relevant social work, counseling, or medical experts for further physical and psychological assessment and treatment (18).

A culture of safety in sports promotes and reinforces trauma-informed care in sports in several ways; including athlete victims (survivors) of abuse in education initiatives to prevent maltreatment in sports has proven beneficial due to their lived experiences and intricate understanding of abuse within the sports culture (1, 11, 18). Trauma-informed education practices also provide knowledge of signs and symptoms of maltreatment, which holds a broader relevance for others who may be experiencing trauma from something other than abuse, such as the loss or death of a family member, parental separation, critical injury, prolonged COVID-19 lockdowns and racism (59, 60). Additionally, using trauma-informed practices and education with stakeholders and athletes provides education about abuse, which decreases the likelihood of re-traumatization and provides appropriate attention to practices and procedures in a culturally sensitive way which further promotes the ideologies of a culture of safety vs. a culture that promotes winning at all costs (1).

## Conclusion

Ignoring the epidemic of maltreatment in sports could be detrimental to what sports can positively do for an athlete's wellbeing and the development of healthy coping skills. Successful prevention and eradication of maltreatment in sports must be a multiagency, systematic approach involving athletes, entourage members, coaches, sport managers, team physicians, trainers, educators, and fans. Athletes should also be empowered to use their voice and provided avenues to speak up and advocate for positive changes for future generations of athletes.

While there has been significantly more research on relational forms of maltreatment in sports over the last decade, minimal research is available on non-relational forms of maltreatment, including athlete exploitation, athlete trafficking, and organizational abuse rooted in systematic maltreatment. Additional research should focus on developing holistic approaches to combat maltreatment, such as athlete-centered coaching models and education for all stakeholders on the signs and symptoms of abuse (6).

Although several laws exist to prevent sexual abuse in sports and on school campuses, minimal interventions and policies for other forms of non-relational maltreatment in sports have been explored. International mandates on prohibiting the sexual relationships between athletic staff members, stakeholders, and athletes; reporting and investigating requirements of suspected or reported abuse; and the use of third-party entities to promote athlete autonomy and hold organizations accountable for potential maltreatments, whether relational or non-relational, should all be further explored for the advocation of athletes and the prevention of maltreatment in sports.

The importance of a trauma-informed perspective in advocating for athletes and promoting a culture of safety in sports cannot be understated, as seen by the documented biopsychosocial impacts of maltreatment on long-term health and wellbeing (61). In reality, the effects impacting athletes discussed in this conceptual analysis affect the broader population who also experience maltreatment and thus show the importance of implementing trauma-informed perspectives in sports at every age group and level (62). Maltreatment and trauma can be perpetrated on athletes in relational and non-relational ways, drastically broadening the need for future research on maltreatment in sports. Individuals, organizations, and systematic policies and practices can all perpetrate abuse on athletes. Individuals and teams can be affected by such maltreatment, and ensuring the correct professionals, like sports social workers, are included as part of the interdisciplinary team is imperative for ensuring proper care is provided and biases toward the organizations or coaches do not hinder reporting or investigating allegations of maltreatment.

## Author contributions

All authors listed have made a substantial, direct, and intellectual contribution to the work and approved it for publication.

## Conflict of interest

The authors declare that the research was conducted in the absence of any commercial or financial relationships that could be construed as a potential conflict of interest.

## Publisher's note

All claims expressed in this article are solely those of the authors and do not necessarily represent those of their affiliated organizations, or those of the publisher, the editors and the reviewers. Any product that may be evaluated in this article, or claim that may be made by its manufacturer, is not guaranteed or endorsed by the publisher.
